# Effect of a Comprehensive Mobile-Based Respiratory Training Program on Respiratory Function in Survivors of Acute Stroke: Randomized Controlled Trial

**DOI:** 10.2196/78637

**Published:** 2026-04-14

**Authors:** Zong-Ke Ma, Han-Hong Jiang, Yan-Hua Tang, Jia Yang, Qing-Chuan Wei, Qiang Gao

**Affiliations:** 1 Rehabilitation Medicine Center and Institute of Rehabilitation Medicine, West China Hospital of Sichuan University, No.37 Guoxue Lane, Wuhou District, Chengdu, 610041, China, +86-28-85422847; 2 Key Laboratory of Rehabilitation Medicine in Sichuan Province, West China Hospital of Sichuan University, Chengdu, China; 3 Department of Rehabilitation Medicine, Sichuan Mianyang 404 Hospital, Mianyang, China

**Keywords:** acute stroke, mobile-based, respiratory function, respiratory muscle training, functional capacity

## Abstract

**Background:**

Respiratory dysfunction frequently occurs during the acute phase of stroke and is associated with reduced ventilatory capacity, respiratory muscle weakness, and increased pulmonary complications. However, delivering standardized respiratory training during hospitalization is often constrained by staffing and service continuity.

**Objective:**

This study aimed to evaluate the efficacy, safety, and feasibility of a hospital-based comprehensive mobile-based respiratory training program (CMRTP) added to conventional rehabilitation in people with acute stroke who are inpatients.

**Methods:**

This single-center, assessor-blinded randomized controlled trial enrolled 40 patients within 2 weeks after stroke onset with respiratory dysfunction (forced vital capacity <80% predicted). Participants were randomized (1:1) to CMRTP plus conventional rehabilitation or conventional rehabilitation alone. The CMRTP was delivered via the WeChat-based AIRHUB platform and performed 20 minutes twice daily, 5 days per week for 2 weeks, either independently or with caregiver assistance as needed. The primary outcome was change in forced vital capacity from baseline to week 2. Secondary outcomes included forced expiratory volume in 1 second (FEV₁), peak expiratory flow, maximal inspiratory pressure, maximal expiratory pressure, and modified Barthel index. All outcomes were assessed face-to-face by a blinded senior physician, and all analyses followed an intention-to-treat principle.

**Results:**

Of 56 screened patients, 40 were randomized, and 39 completed the study. Adherence to the CMRTP reached 96%, and no serious adverse events occurred; mild, transient events (fatigue, dizziness, and hyperventilation) were recorded. Compared with the control group, the CMRTP group demonstrated greater improvement in forced vital capacity at week 2 (mean difference 0.77 L; 95% CI 0.39‐1.16; *P*<.001; η²=0.32), with additional between-group differences in maximal inspiratory pressure (*P*=.001; η²=.25), maximal expiratory pressure (*P*<.001; η²=.08), and modified Barthel index (*P*=.001; η²=.26). No significant group differences were found for forced expiratory volume in 1 second or peak expiratory flow.

**Conclusions:**

A 2-week hospital-based mobile respiratory training program is feasible and safe in people with acute stroke who are inpatients and yields clinically meaningful improvements in respiratory function and daily functional performance when added to conventional rehabilitation.

## Introduction

Stroke is the second-leading cause of death and the third-leading cause of disability worldwide, imposing a substantial burden on society and families [1]. Advances in acute stroke management have improved survival rates; however, most survivors experience persistent functional impairments [2]. In addition to motor, sensory, swallowing, and speech impairments, stroke frequently induces respiratory dysfunction during the acute stage [3]. Recent studies indicate that 18% to 88% of patients with stroke exhibited abnormal breathing patterns [4], while 44% to 90% present with respiratory failure syndrome of varying severity [5]. Furthermore, 7% to 38% develop pneumonia within the first week after the onset of acute stroke [6], underscoring the urgent need for effective interventions to improve respiratory function.

Clinical guidelines advocate early respiratory monitoring and intervention to mitigate poststroke respiratory complications, emphasizing an integrated care that includes physical and respiratory rehabilitation [7]. Previous studies have demonstrated that respiratory muscle training and breathing exercises, such as diaphragmatic, air-stacking, and pursed-lip breathing, can improve respiratory muscle strength, pulmonary function, and functional capacity during early stroke rehabilitation [8-12]. However, the implementation of respiratory interventions in stroke rehabilitation remains limited by multiple clinical constraints, including insufficient staffing and inconsistent service delivery [13-16]. In many health care settings, rehabilitation services are delayed or underused due to the prioritization of acute medical management, leaving survivors with stroke with inadequate support for respiratory dysfunction [17-19]. In this context, telerehabilitation may represent a promising alternative for delivering timely and accessible respiratory rehabilitation interventions.

Telerehabilitation has emerged globally as a promising approach to improving access to and continuity of stroke rehabilitation services, attracting widespread attention [20,21]. Chen et al [22] reported that telerehabilitation has been widely applied in stroke rehabilitation and provided effects comparable to conventional rehabilitation in improving motor function among survivors with stroke. Linder et al [23] demonstrated that mobile-based exercise interventions can effectively enhance quality of life and alleviate depressive symptoms among individuals recovering from stroke. In addition, Sun et al  [24] found that telerehabilitation can effectively reduce caregiver burden. Moreover, a growing body of evidence from high-income countries supports the feasibility, safety, and effectiveness of telerehabilitation in the postacute and chronic phases of stroke recovery [25-27]. However, despite these encouraging findings, the application of telerehabilitation during the acute hospitalization phase of stroke remains largely unexplored.

Therefore, this study aimed to evaluate the effectiveness of a 2-week hospital-based comprehensive mobile-based respiratory training program (CMRTP) on respiratory function in patients with acute stroke, and to explore its safety and feasibility for early inpatient rehabilitation. We hypothesized that integrating CMRTP with conventional rehabilitation would yield greater improvements in respiratory function, without increasing the incidence of adverse events.

## Methods

### Design

We conducted an assessor-blinded, parallel group, randomized controlled trial at Sichuan Mianyang 404 Hospital (Mianyang, Sichuan, China) from September 2024 to March 2025. This study was prospectively registered with the Chinese Clinical Trial Registry (ChiCTR2400088647). The study adhered to the CONSORT (Consolidated Standards of Reporting Trials) statement guidelines (Checklist 1) [28], and all procedures complied with the Declaration of Helsinki.

### Participants

Participants were recruited from the inpatient departments of Rehabilitation Medicine, Neurology, and Neurosurgery through electronic medical records screening. A physician (GQ) screened potential participants based on the eligibility criteria.

Participants were eligible if they met the following criteria: (1) aged 18‐75 years; (2) diagnosed with stroke according to the Diagnostic Criteria of Cerebrovascular Diseases [29]; (3) first-ever unilateral ischemic stroke confirmed by neuroimaging; (4) stroke onset within 2 weeks; (5) respiratory dysfunction, defined as forced vital capacity (FVC) <80% predicted value [30]; and (6) either the participants or their caregivers were proficient in reading Chinese characters and in operating a smartphone or tablet.

Participants were excluded if they met any of the following criteria: (1) cognitive deficits (Mini-Mental Score Examination scores for patients with stroke: illiterate <17 points, primary school education <20 points, and junior high school education and above <24 points) or inability to understand the instructions provided by researchers; (2) having other diseases, surgeries, or injuries that may interfere with respiratory function training (like severe lung infections, tracheostomy intubation, myasthenia gravis, phrenic nerve paralysis, severe facial paralysis, oropharyngeal structural abnormalities, a history of chest or abdominal surgery or rib fractures within the past year); or (3) refuse or withdraw from this research.

### Randomization and Blinding

Patients were randomly assigned in a 1:1 ratio to either the experimental group (the CMRTP plus conventional rehabilitation) or control group (conventional rehabilitation only) using a computer-generated randomization sequence (IBM SPSS version 29). Allocation concealment was ensured by a research assistant, who was not involved in sequence generation or participant enrollment, using sequentially numbered, sealed, opaque envelopes. Each envelope was opened only after a participant had been formally enrolled, and the assigned intervention was then revealed. Outcome assessors and data analysts were blinded to group allocation throughout the trial.

### Intervention

#### Overview

Patients in the experimental group received the CMRTP plus conventional rehabilitation, while the control group received conventional rehabilitation alone, without specific respiratory training. Conventional rehabilitation was administered by two licensed physiotherapists for 40 minutes per session, once daily, 5 days per week, over a 2-week period. The CMRTP was delivered via the AIRHUB platform (a mobile WeChat-based applet, AIRHUB TECH) by two licensed physiotherapists (JY and Z-KM), with 20 minutes per session, twice daily, 5 days per week for 2 weeks.

#### The CMRTP Intervention

**Figure 1.**
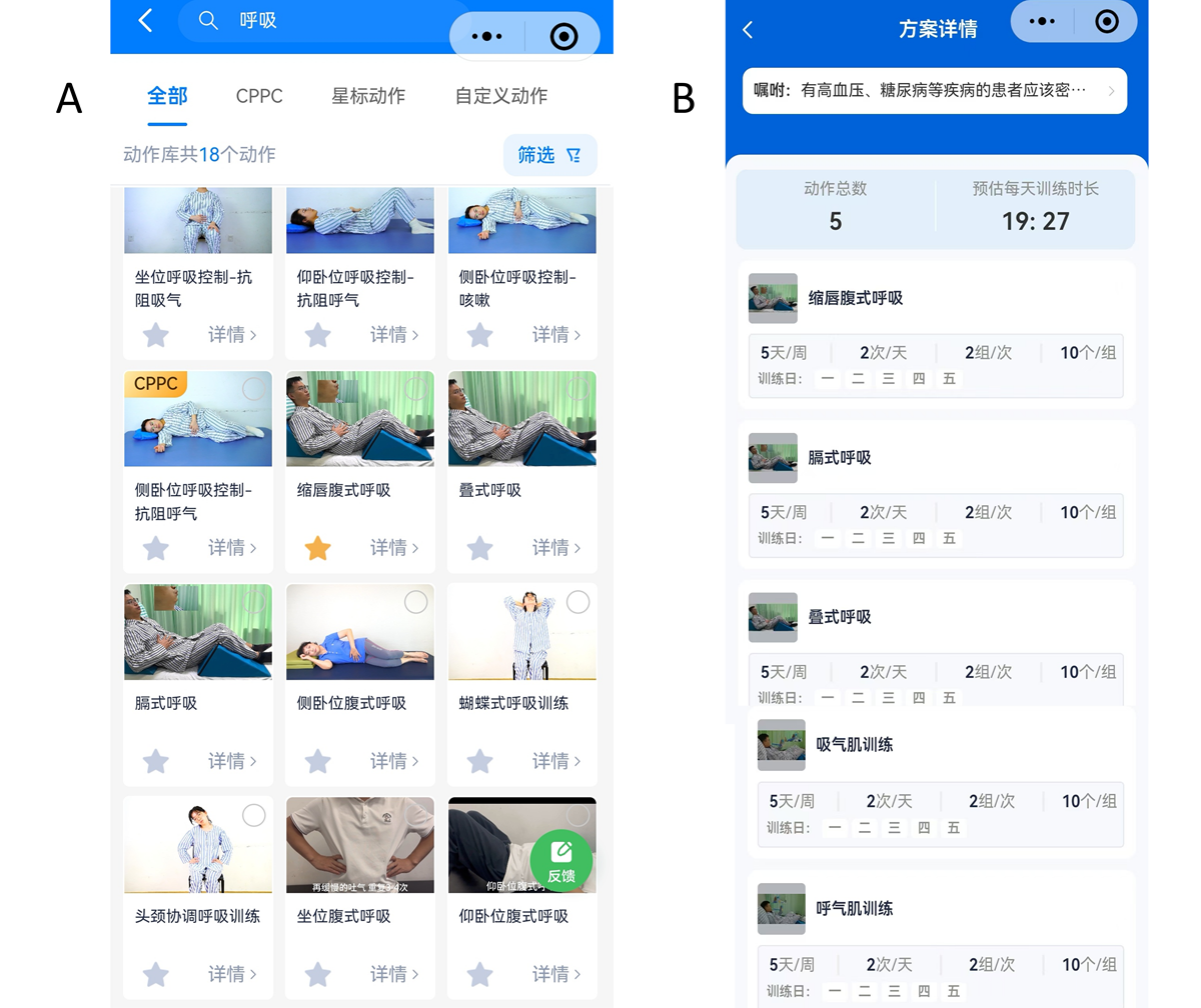
Therapist interface of the AIRHUB platform. (A) Video library and (B) comprehensive mobile-based respiratory training program.

**Figure 2.**
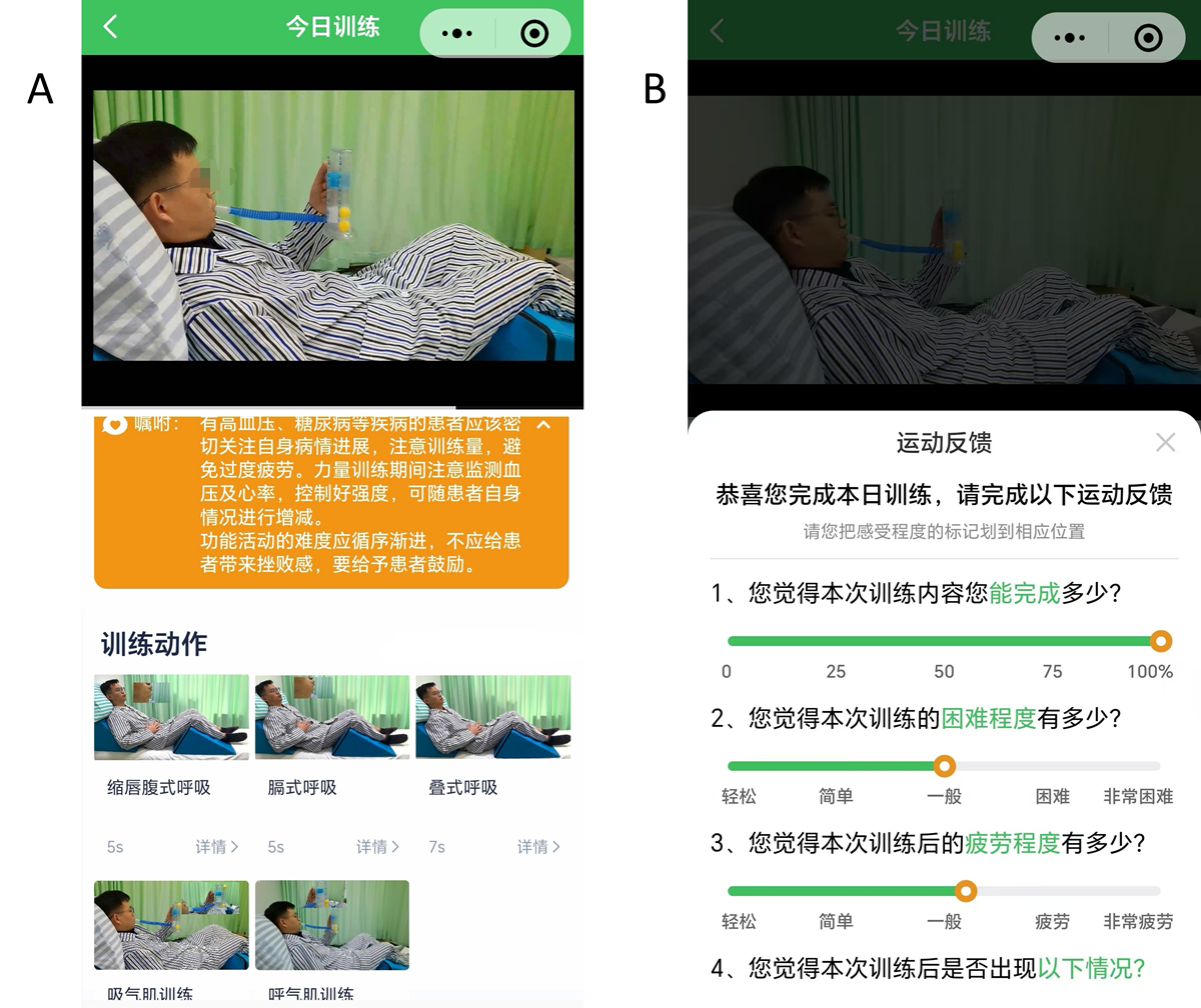
Patient interface of the AIRHUB platform (A: exercise plan; B: feedback submission).

The AIRHUB platform is a WeChat Applet–based telerehabilitation system consisting of a cloud-based server, a therapist interface (Figure 1), and a patient interface (Figure 2). The therapist interface provides access to an exercise video library, remote prescription and monitoring modules, and real-time feedback collection, while the patient interface allows video-guided exercises, automatic performance recording, and submission of postsession feedback. All data are synchronized securely to the cloud, enabling therapists to review adherence and feedback. The platform supports multiuser access and modular expansion, allowing integration with other rehabilitation programs and scalability to multicenter use. To protect privacy, all patient data are encrypted, deidentified, and accessible only to authorized personnel, in accordance with data protection standards aligned with the principles of the HIPAA (Health Insurance Portability and Accountability Act).

In the experimental group, two licensed physiotherapists (YJ and Z-KM) provided guidance to patients or their caregivers on the proper use of the AIRHUB platform for 2 hours, including the login process, conducting respiratory training, and performing movements correctly as demonstrated in the instructional videos prior to the intervention. After confirming that participants had achieved adequate training proficiency, the physiotherapists guided them to register a personal account on the patient interface using their own mobile phone number. The physiotherapist then logged into the therapist interface, selected the corresponding CMRTP videos from the motion library, and sent them to the individual patient interface.

**Figure 3.**
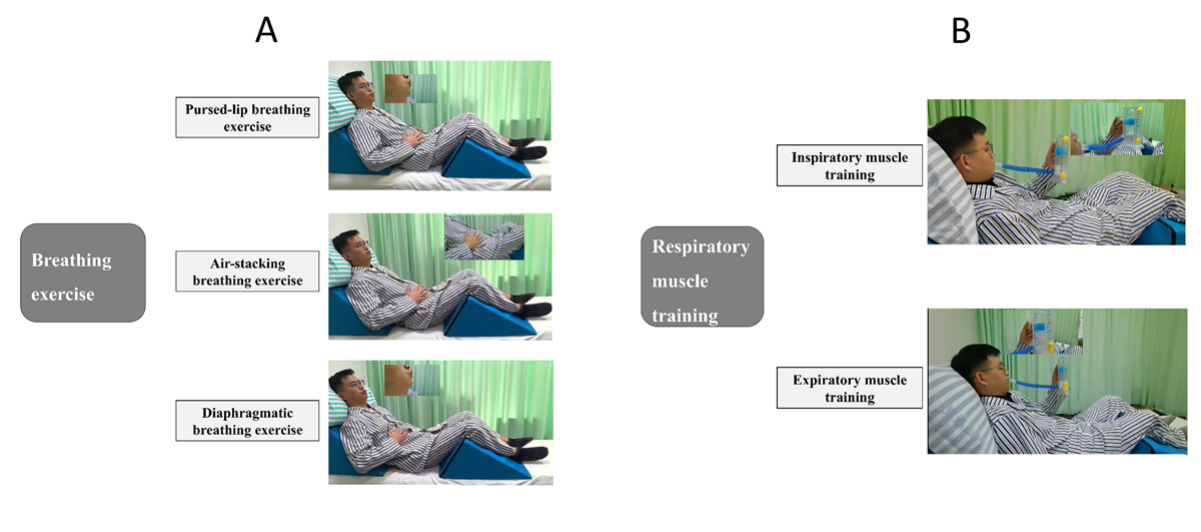
Breathing exercises and respiratory muscle training in AIRHUB. (A) Breathing exercises and (B) respiratory muscle training.

The CMRTP consisted of 5 exercises, including breathing exercises and respiratory muscle training (Figure 3; details are mentioned in Multimedia Appendix 1), each accompanied by audio cues and written instructions. Each exercise was performed 5 times, with a 20-second rest interval between each exercise. After completing all exercises, the entire plan was repeated once. During the intervention, participants accessed the CMRTP via the patient interface, where they followed standardized demonstration videos and audio instructions to perform the exercises synchronously, and the playback speed could be adjusted as needed. Upon completion, the patient provided feedback through a platform-based questionnaire, and the therapist supervises the training remotely via WeChat or mobile video calls as they needed.

To ensure adherence and track progress, two licensed physiotherapists (YJ and Z-KM) reviewed the training status, such as session duration and questionnaire responses, three times weekly via the AIRHUB platform to evaluate overall completion. When potential issues were identified during monitoring, the responsible physiotherapist would promptly reach out to the participant and offer support. Moreover, the primary nurse provided supervision and reminders to patients and caregivers. If a patient did not engage for three consecutive days, the system automatically triggered a SMS text message reminder. In cases where no training activity was recorded thereafter, the physiotherapist conducted a follow-up telephone call to identify barriers and encourage continued participation.

### Conventional Rehabilitation

Conventional rehabilitation [8] was provided by 2 licensed physiotherapists (QCW and HHJ), including the following exercises: (1) joint range of motion exercises in bed, (2) maintaining proper limb positioning in bed, (3) turning exercises, (4) positional transfer training, (5) sitting exercises in bed, and (6) standing exercise (if feasible). If necessary, occupational and speech therapy may also be included.

### Assessments and Outcomes

All baseline evaluations were independently performed by a research coordinator after participant enrollment. Primary and secondary outcomes were assessed at baseline (T0), week 1 (T1), and week 2 (T2) following randomization. Outcome assessments were completed face-to-face by a senior physician (HHJ).

### Primary Outcome

The primary outcome was the change in FVC from baseline to week 2. FVC, defined as the total volume of air exhaled during a maximal forced expiration following a full inspiration, was used to evaluate overall improvements in respiratory function for patients with acute stroke [31,32]. The minimal clinically important difference for FVC was preset at a 5% predicted increase [33,34]. FVC was measured in liters using standardized spirometry (Breath Home, Home Sun Int.) in accordance with American Thoracic Society and European Respiratory Society standard guidelines [35-38]. FVC was measured three times per assessment, with the highest value used for analysis.

### Secondary Outcomes

The secondary outcomes included forced expiratory volume in 1 second (FEV₁) and peak expiratory flow (PEF) to assess airflow dynamics [39], maximal inspiratory pressure (MIP) and maximal expiratory pressure (MEP) to measure respiratory muscle strength [40], and the modified Barthel Index (MBI) to evaluate functional performance in daily activities [41]. Feasibility (adherence rate) and safety (adverse events) were also examined as secondary endpoints. FEV₁, PEF, MIP, and MEP were measured using the same spirometry device (Breath Home), and MBI was assessed using a validated scale by the same physician following standardized procedures.

### Sample Size

The sample size calculation was conducted via G*power of 3.1.9.7 based on the result of the FVC in a published similar study [33,42], which indicated an estimated effect size of *f*=0.27. Other parameters were set as follows: a significance level of α=.05 (two tails), power (1–β)=95%, correlation among repeated measures=0.5, nonsphericity correction *ε*=1, number of measurements=3, and number of groups=2. Therefore, a sample size of n=32 was obtained. After allowing for a 20% attrition, a minimum total of 40 participants was needed.

### Statistical Analysis

Baseline assessments included patient characteristics, FVC, FEV₁, PEF, MIP, MEP, and MBI. All data were analyzed using an intention-to-treat approach, with missing values imputed via the last observation carried forward method.

Statistical analyses used IBM SPSS version 29 (IBM Corp). The Shapiro-Wilk test assessed data normality, and the Levene test was applied to assess the homogeneity of variances. Continuous variables were reported as mean and SD values, ordinal variables as median with IQR values, and categorical variables as numbers with percentages. Baseline characteristics were compared using *t* tests for normally distributed continuous variables, Mann-Whitney *U* tests for nonnormally distributed or ordinal variables, and chi-square tests for categorical variables. Repeated measures analysis of covariance (ANCOVA) evaluated between-group differences across time points, adjusting for baseline values, with least squares mean differences (least squares [LS] mean differences) estimated to quantify treatment effects. Fixed effects included group, time, and time× group interaction, with participant and National Institutes of Health Stroke Scale (NIHSS) as a random effect. The Mauchly test assessed sphericity, with Greenhouse-Geisser correction applied if violated. Bonferroni correction was applied only to unplanned post hoc pairwise comparisons. The primary outcome was not adjusted for multiple comparisons. For secondary outcomes, Bonferroni correction was applied to control the risk of type I error. Significance was set at *P*<.05 (2-tailed) for the primary outcome and α<.025 (2-tailed) for others.

### Ethical Considerations

The study was approved by the Ethics Committee of Sichuan Mianyang 404 Hospital (ethics reference: 2023‐051). Written informed consent was obtained from all participants prior to enrollment after they had received a full explanation of the study procedures. All data were anonymized to protect participant privacy and confidentiality. No financial compensation was provided to participants. To ensure participants’ safety, all potential adverse events were predefined and explicitly described in the informed consent form. Adverse events were monitored by the physiotherapists responsible for the intervention in the experimental group. Potential adverse events associated with tele-respiratory training included fatigue, tiredness, dizziness, headache, falls, worsening neurological function, hyperventilation, hypoventilation, decreased oxygen saturation, hypertension, and tachycardia. If any adverse events occur during the intervention, caregivers or primary nurses would immediately inform the patient’s attending physician and take appropriate measures. All adverse events associated with conventional rehabilitation or CMRTP were recorded on the case report form for final analysis.

## Results

**Figure 4.**
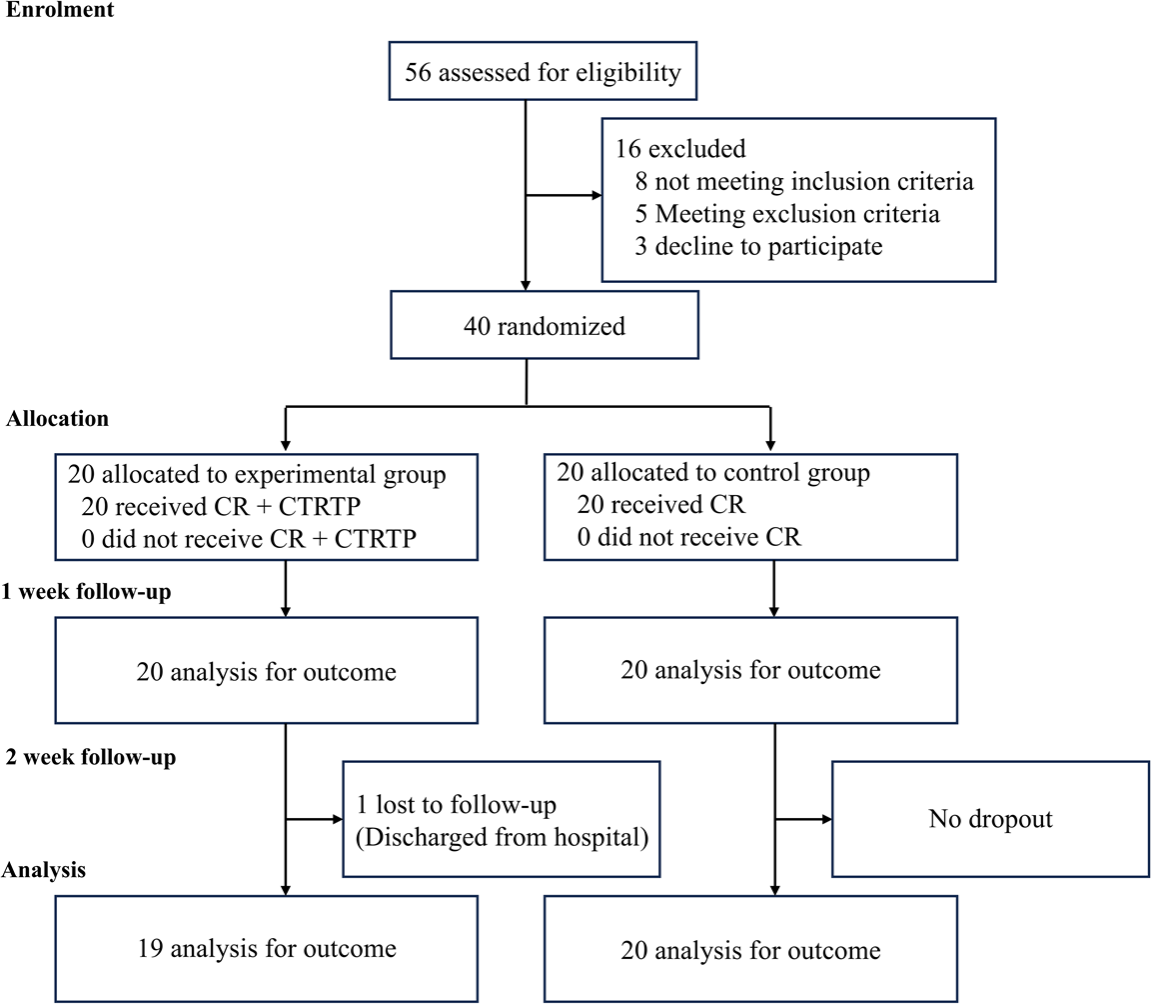
The CONSORT (Consolidated Standards of Reporting Trials) flow diagram of the trial. CR: conventional rehabilitation; CMRTP: comprehensive mobile-based respiratory training program.

A total of 56 patients were screened for eligibility, and 40 were enrolled in the study. Of these, 20 were randomly assigned to the intervention group and 20 to the control group (Figure 4). One participant in the intervention group withdrew during the second week due to hospital discharge. Regarding treatment adherence, participants in the experimental group completed 192 of the 200 prescribed training sessions (96%), whereas those in the control group completed all 100 assigned sessions (100%). Outcome data for analysis were available for 100% (N=40) of participants at T1 and 97.5% (39/40) at T2. Nonserious adverse events considered reasonably or definitely related to study procedures were recorded in 10 sessions in the experimental group (including fatigue, dizziness, and hyperventilation) and in 3 sessions in the control group (including fatigue and tiredness). All events were transient and mild, and no serious adverse events occurred in either group.

The baseline clinical characteristics of the patients, shown in Table 1, were generally comparable between the experimental and control groups. All included patients had moderate stroke with notable functional impairments affecting daily activities. The mean (SD) age was 62.5 (SD 10.57) years. The experimental group exhibited slightly greater stroke severity than the control group (mean NIHSS score 7.30, SD 1.75 vs 5.75, SD 1.97; *P=*.01), while other clinical features were comparable between groups.

**Table 1.** Clinical characteristics of the patients at baseline.

Characteristics	Intervention (n=20)	Control (n=20)	Test statistic	*P* value
Gender, n (%)			0.45 (1.00)^a^	.50
Male	15 (75)	12 (60)		
Female	5 (25)	8 (40)		
Age (years), mean (SD)	59.80 (11.31)	65.20 (9.83)	1.61 (37.29)^b^	.12
Course of disease (day), mean (SD)	6.00 (2.17)	6.20 (2.31)	0.28 (37.87)^b^	.78
BMI^c^ (kg/m^2^), mean (SD)	25.25 (2.57)	24.48 (3.45)	0.80 (35.12)^b^	.43
NIHSS^d^^,^^e^	7.30 (1.75)	5.75 (1.97)	2.63 (37.48)^b^	.01
MMSE^f^	25.35 (1.89)	25.05 (1.79)	0.56 (37.79)^b^	.58
Stroke type, n (%)			0.27 (1.00)^a^	.59
Ischemic stroke	19 (95)	17 (85)		
Hemorrhagic stroke	1 (5)	3 (15)		
Affected side, n (%)			0.00 (1.00)^a^	>.99
Left	13 (65)	13 (65)		
Right	7 (35)	7 (35)		
Coexisting conditions, n (%)				
Hypertension	14 (70)	15 (75)	0.00 (1.00)^a^	>.99
Diabetes mellitus	5 (25)	9 (45)	0.98 (1.00)^a^	.32
Pulmonary infection	0 (0)	1 (5)	0.00 (1.00)^a^	>.99
COPD^g^	2 (10)	0 (0)	0.52 (1.00)^a^	.46
Smoking	0 (0)	1 (5)	0.10 (1.00)^a^	.75

a Chi-square (df).

b *t* test (df).

c The BMI is the weight in kilograms divided by the square of the height in meters.

d Independent-samples *t* tests (*P*<.05).

e NIHSS: National Institutes of Health Stroke Scale.

f MMSE: Mini-Mental State Examination.

g COPD: chronic obstructive pulmonary disease.

**Figure 5.**
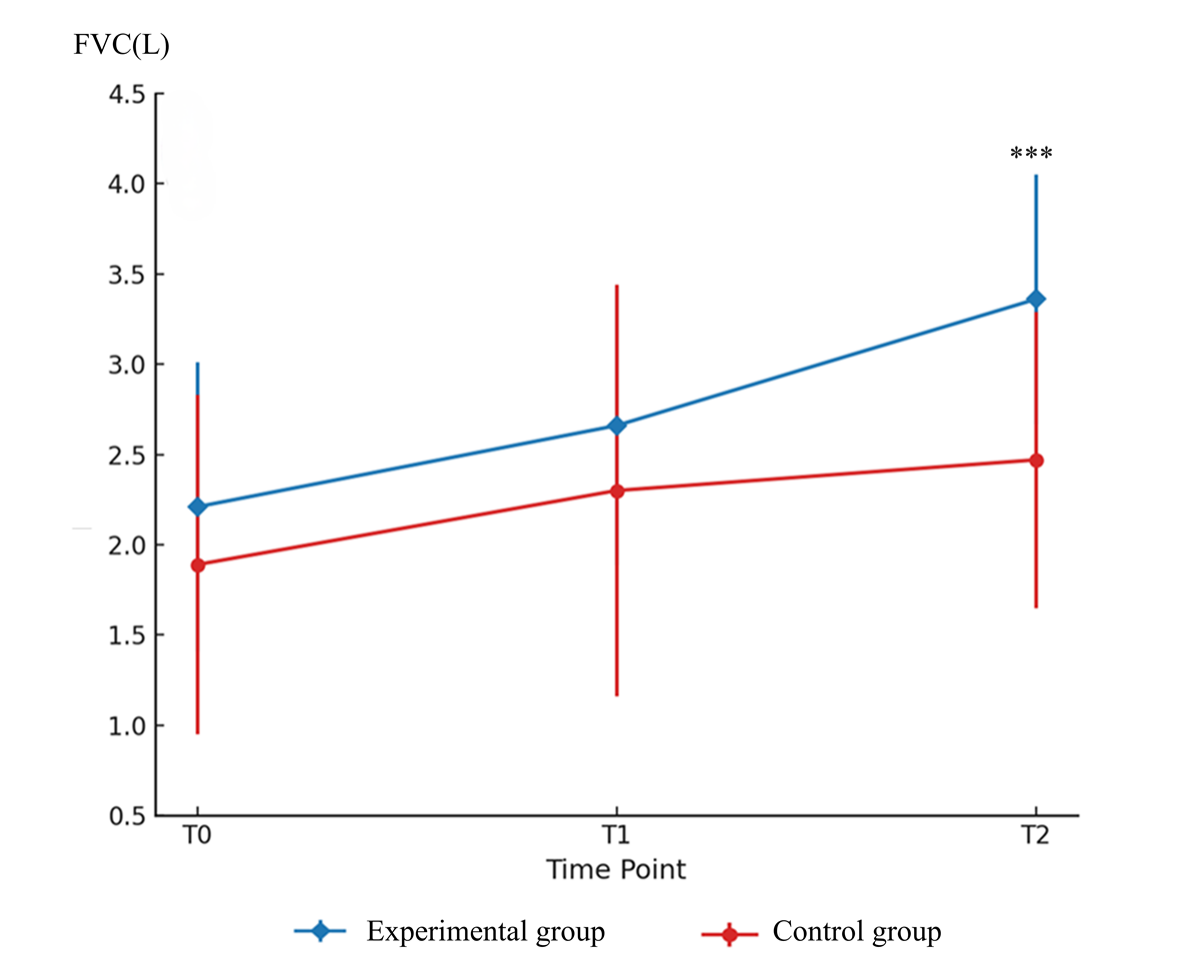
Between-group temporal changes in forced vital capacity. Mean (± SD) values are shown at baseline (T0), week 1 (T1), and week 2 (T2). The experimental group exhibited greater improvement than the control group at T2 (***P<.001). FVC: forced vital capacity.

For the primary outcome, the analysis of FVC revealed significant main effects in both the time (*F*_1,37_=41.12; η²=0.52; *P*<.001) and time×group interaction effect (*F*_1,37_=4.50; η²=0.12; *P*=.008), without group effect (*F*_1,37_=4.05; η²=0.10; *P*=.05; Table 2). The FVC at week 2 increased by 1.12 L (95% CI 0.43‐1.81) in the experimental group and 0.58 L (95% CI 0.02‐1.14) in the control group, a significant difference was observed between the groups at week 2 (LS mean 0.77, 95% CI 0.39‐1.16; η²=0.32; *P*<.001; Figure 5). The improvements of both groups exceeded the minimal clinically important difference (5%), but the experimental group showed a greater improvement (Table 3).

**Table 2.** Summary of main effects and interaction effects from repeated measures analysis of covariance.

Outcome	*Group* *effect, F *test *(df)*	*P* value (Group)	η^2^ (Group)	Time effect, *F test* (*df*)	*P* value (Time)	η^2^ (Time)	Group×time interaction, *F* test (*df*)	*P* value (Interaction)	η^2^ (Interaction)
FVC^a^ (L)	4.05 (1,37)	.05	0.10	41.12 (1,37)	＜.001^b^	0.52	4.50 (1,37)	.008^c^	0.12
FEV₁^d^ (L)	2.21 (1,37)	.15	0.06	6.86 (0.83,30.71)	.004^c^	0.16	1.25 (0.83,30.71)	.29	0.03
PEF^e^ (L/s)	1.89 (1,37)	.18	0.05	1.37 (0.83,30.71)	.008^c^	0.14	1.66 (0.83,30.71)	.20	0.04
MIP^f^ (cmH₂O)	14.49 (1,37)	＜.001^b^	0.28	7.66 (1,37)	＜.001^b^	0.17	7.28 (1,37)	.001^c^	0.16
MEP^g^ (cmH₂O)	10.54 (1,37)	.002^c^	0.20	1.37 (0.83,30.71)	.26	0.04	4.62 (0.83,30.71)	.02^h^	0.11
MBI^i^ (score)	7.56 (1,37)	.009^c^	0.17	10.07 (1,37)	＜.001^b^	0.21	3.35 (1,37)	.04^h^	0.08

a FVC: forced vital capacity.

b *P*<.001.

c *P*<.01.

d FEV₁: forced expiratory volume in 1 second.

e PEF: peak expiratory flow.

f MIP: maximal inspiratory pressure.

g MEP: maximal expiratory pressure.

h *P*<.05.

i MBI: modified Barthel index.

**Table 3.** Analysis of the effect of time and group on the primary and secondary outcomes.

Test and groups	T0^a^	T1^b^^c^	T2^b^^d^	ΔT1^e^	ΔT2^f^	*F*	*P* value	η^2g^
FVC^h^ (L)								
Experimental group (n=20), mean (SD)	2.21 (0.80)	2.66 (0.77)^i^	3.36 (0.69)^i^	0.45 (0.27)	1.12 (0.69)	38.31	<.001^j^	0.67
Control group (n=20), mean (SD)	1.89 (0.94)	2.30 (1.14)	2.47 (0.82)^i^	0.41 (0.73)	0.58 (0.56)	9.59	.001^k^	0.34
LS^l^ means (95% CI)	0.32 (–0.24 to 0.88)	—^m^	—	0.05 (–0.36 to 0.46)	0.77 (0.39‐1.16)	—	—	—
*t (df)*	1.15 (38)	—	—	0.27 (24.03)	4.08 (38)	—	—	—
*P*^n^	0.26	—	—	0.79	<.001^j^	—	—	—
η^2^	0.03	—	—	0.002	0.32	—	—	—
FEV₁^o^ (L)								
Experimental group (n=20), mean (SD)	1.41 (0.55)	1.77 (0.81)	2.44 (0.87)^i^	0.36 (0.71)	1.41 (0.55)	21.48	<.001^j^	0.53
Control group (n=20), mean (SD)	1.38 (0.71)	1.35 (0.72)	2.04 (0.74)^i^	–0.03 (0.88)	0.66 (0.69)	10.74	<.001^j^	0.36
LS means (95% CI)	0.10 (–0.35 to 0.55)	—	—	0.28 (–0.28 to 0.84)	0.36 (–0.15 to 0.88)	—	—	—
*t (df)*	0.45(38)	—	—	1(38)	1.44(38)	—	—	—
*P*^n^	0.65	—	—	0.33	0.16	—	—	—
η^2^	0.006	—	—	0.03	0.05	—	—	—
PEF^p^ (L/s)								
Experimental group (n=20), mean (SD)	2.40 (1.08)	2.81 (0.95)	3.71 (1.20)^i^	0.42 (0.84)	1.23 (1.18)	19.09	＜.001^j^	0.5
Control group (n=20), mean (SD)	2.34 (1.32)	2.50 (1.19)	2.93 (0.99)	0.16 (1.29)	0.59 (1.14)	2.99	0.07	0.14
LS means (95% CI)	0.20 (–0.65 to 1.05)	—	—	0.27 (–0.37 to 0.92)	0.73 (0.04‐1.42)	—	—	—
*t (df)*	0.48 (38)	—	—	0.85 (38)	2.14 (38)	—	—	—
*P*^n^	0.64	—	—	0.4	0.04	—	—	—
η^2^	0.006	—	—	0.11	0.11	—	—	—
MIP^q^ (cmH₂O)								
Experimental group (n=20), mean (SD)	34.45 (15.64)	45.62 (15.93)^i^	51.84 (15.06)^i^	11.17 (7.10)	16.55 (10.43)	41.48	＜.001^j^	0.69
Control group (n=20), mean (SD)	26.50 (11.82)	29.40 (17.42)	35.01 (14.52)^i^	2.90 (10.71)	8.51 (7.64)	8.5	＜.001^j^	0.31
LS means (95% CI)	11.16 (1.75‐20.56)	—	—	10.83 (4.12‐17.54)	11.38 (4.69‐18.08)	—	—	—
*t (df)*	2.4 (38)	—	—	3.27 (38)	3.45 (38)	—	—	—
*P*^n^	0.08	—	—	.002	.001**^k^	—	—	—
η^2^	0.07	—	—	0.23	0.25	—	—	—
MEP^r^ (cmH₂O)								
Experimental group (n=20), mean (SD)	37.34 (18.98)	47.85 (24.00)^i^	56.28 (20.15)^i^	10.51 (13.99)	17.83 (12.24)	21.95	＜.001^j^	0.53
Control group (n=20), mean (SD)	32.94 (20.26)	35.29 (25.65)	36.25 (16.01)	2.36 (20.46)	3.32 (11.24)	0.48	0.55	0.03
LS means (95% CI)	11.49 (–1 to 23.98)	—	—	11.68 (–1.18 to 24.54)	17.85 (9.92‐25.77)	—	—	—
*t (df)*	1.86 (38)	—	—	1.84 (38)	4.57 (38)	—	—	—
*P*^c^	0.07	—	—	0.07	＜.001^j^	—	—	—
η^2^	0.09	—	—	0.09	0.08	—	—	—
MBI^s^ (score)								
Experimental group (n=20), mean (SD)	38.25 (13.47)	53.40 (12.43)^i^	65.15 (10.47)^i^	15.15 (8.75)	26.25 (8.58)	90.42	＜.001^j^	0.83
Control group (n=20), mean (SD)	37.35 (10.23)	48.90 (12.83)^i^	55.85 (11.25)^i^	11.55 (7.47)	18.50 (8.02)	18	＜.001^j^	0.85
LS means (95% CI)	5.18 (–2.44 to 12.80)	—	—	5.77 (0.20‐11.34)	9.22 (3.90‐14.53)	—	—	—
*t (df)*	1.38 (38)	—	—	2.1 (38)	3.52 (38)	—	—	—
*P*^n^	0.18	—	—	0.04	.001^k^	—	—	—
η^2^	0.05	—	—	0.11	0.26	—	—	—

a T0: baseline.

b Within-group comparison in each group with repeated measures analysis of covariance: compare to T0.

c 1T1: week 1.

d T2: week 2.

e ΔT1: the change from T0 to T1.

f ΔT2: the change from T0 to T2.

g η2: partial eta squared.

h FVC: forced vital capacity.

i *P<.05.

j ***P<.001.

k **P < .025.

l LS: least squares.

m Not applicable.

n Comparison between-group difference in change with repeated measures analysis of covariance.

o FEV₁: forced expiratory volume in 1 second.

p PEF: peak expiratory flow.

q MIP: maximal inspiratory pressure.

r MEP: maximal expiratory pressure.

s MBI: modified Barthel index.

**Figure 6.**
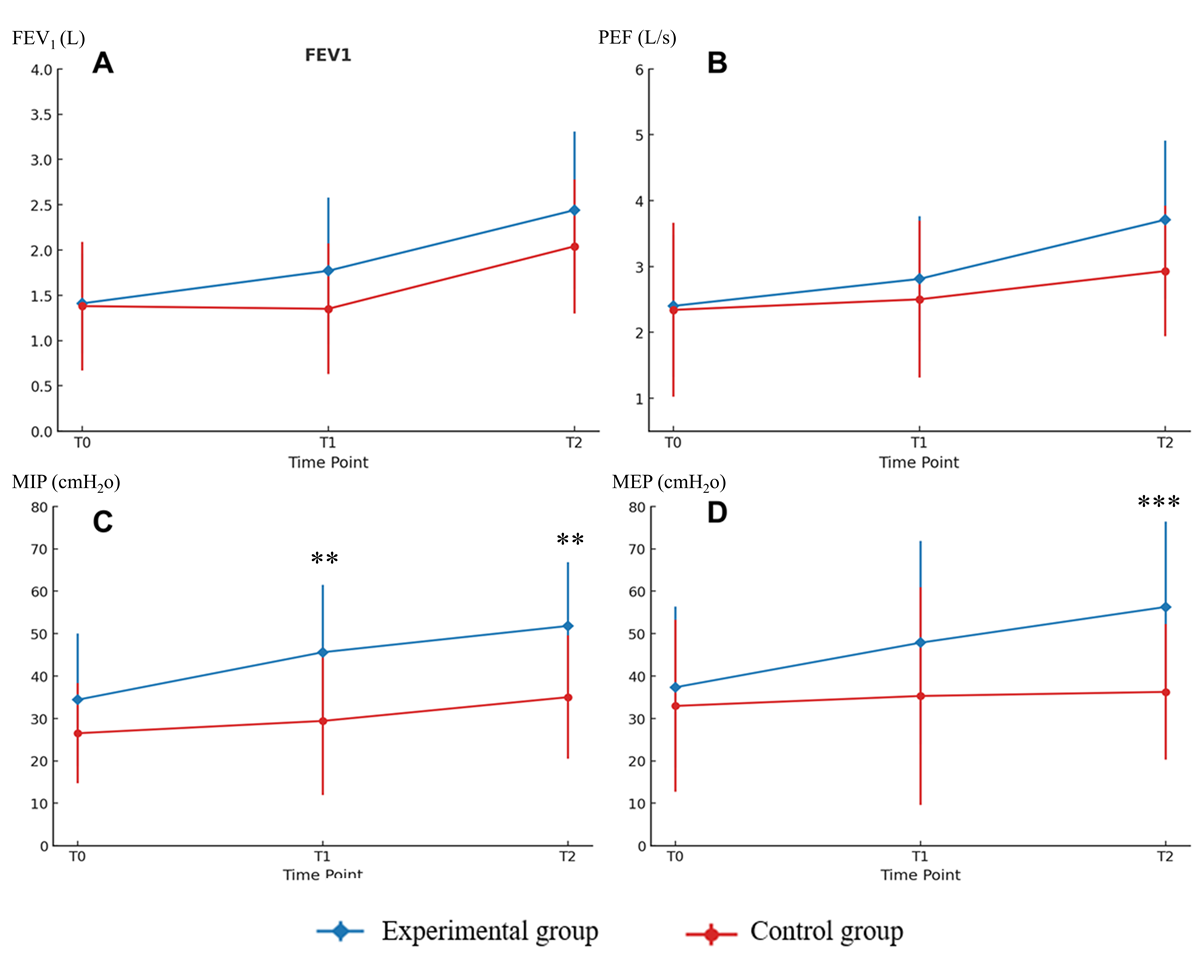
Between-group temporal changes in forced expiratory volume in 1 s, peak expiratory flow, maximal inspiratory pressure, and maximal expiratory pressure. (A) Mean (SD) values are shown at baseline (T0), week 1 (T1), and week 2 (T2). No significant between-group differences were observed in forced expiratory volume in 1 s across all time points. (B) Mean (SD) values are shown at baseline (T0), week 1 (T1), and week 2 (T2). No significant between-group differences were observed in peak expiratory flow across all time points. (C) Mean (SD) values are shown at baseline (T0), week 1 (T1), and week 2 (T2). The experimental group exhibited greater improvement than the control group for maximal inspiratory pressure at T1 (**P=.002) and T2 (**P=.001). D: Mean (SD) values are shown at baseline (T0), week 1 (T1), and week 2 (T2). The experimental group exhibited greater improvement than the control group for maximal expiratory pressure at T2 (***P<.001). FEV1: forced expiratory volume in 1 second; PEF: peak expiratory flow; MIP: maximal inspiratory pressure; MEP: maximal expiratory pressure.

As for secondary outcome in Tables 2 and 3, there was no significant main effect of group for FEV₁ (*F*_1,37_=2.21; η²=0.06; *P*=.15), PEF (*F*_1,37_=1.89; η²=0.05; *P*=.18), whereas MIP (*F*_1,37_=14.49; η²=0.28; *P*<.001), MEP (*F*_1,__37_=10.54; η²=0.20; *P*=.002), and MBI (*F*_1,37_=7.56; η²=.17; *P*=.009) reached statistical significance. The main effect of time for FEV₁ (*F*_0.83,__30.71_=6.86; η²=0.16; *P*=.004), PEF (*F*_0.83,__30.71_=1.37; η²=0.14; *P*=.008), MIP (*F*_1,37_=7.66; η²=0.17; *P*<.001), and MBI (*F_1,37_*=10.07; η²=0.21; *P*<.001) showed significant differences, but not on MEP (*F*_0.76,__28.12_=1.37; η²=0.04; *P*=.26) at weeks 1 and 2. Significant group×time interactions were found for MIP (*F*_1,37_=7.28; η²=0.16; *P*=.001), MEP (*F*_0.76,28.12_=4.62; η²=0.11; *P*=.02), and MBI (*F*_1,37_=3.35; η²=0.08; *P*=.04), but not for FEV₁ (*F*_0.83,__30.71_=1.25; η²=.00; *P*=0.29) and PEF (*F*_0.83,__30.71_=1.66; η²=0.04; *P*=.20). Compared with baseline, the experimental group exhibited significant increases in FEV₁ (*F*_1.66,__31.54_=21.48; η²=0.53; *P*<.001), PEF (*F*_1.66,__31.54_=19.09; η²=0.50; *P*<.001), MIP (*F*_2,__38_=41.48; η²=0.69; *P*<.001), MEP (*F*_1.52,__28.88_=21.59; η²=0.53; *P*<.001), and MBI (*F*_2,__38_=90.42; η²=0.83; *P*<.001), whereas the control group showed significant increases in FEV₁ (*F*_1.66,__31.54_=10.74; η²=0.36; *P*<.001), MIP (*F_2_*_,__38_=8.50; η²=0.31; *P*<.001) and MBI (*F*_2,__38_=90.42; η²=0.85; *P*<.001), but not in PEF (*F*_1.66,__31.54_=2.99, η²=0.14; *P*=.07) and MEP (*F*_1.52,__28.88_=0.48; η²=0.03; *P*=.55). Compared with the control group, the experimental group had no baseline differences (*P*>.05 for FEV₁, PEF, MIP, MEP, and MBI), and showed significant improvements at week 2 in MIP (LS mean 11.38, 95% CI 4.69‐18.08; η²=0.25; *P*=.001), MEP (LS mean 17.85, 95% CI 9.92‐25.77; η²=0.08; *P*<.001), and MBI (LS mean 9.92, 95% CI 3.90‐14.53; η²=0.26; *P*=.001; Figures6  7), as well as in MIP at week 1 (LS mean 10.83, 95% CI 4.12‐17.54; η²=0.23; *P*=.002). No difference was found between the groups in the FEV₁ and PEF at any assessment time point, which may be due to the short intervention duration and the limited sensitivity of these measures to detect subtle respiratory changes.

**Figure 7.**
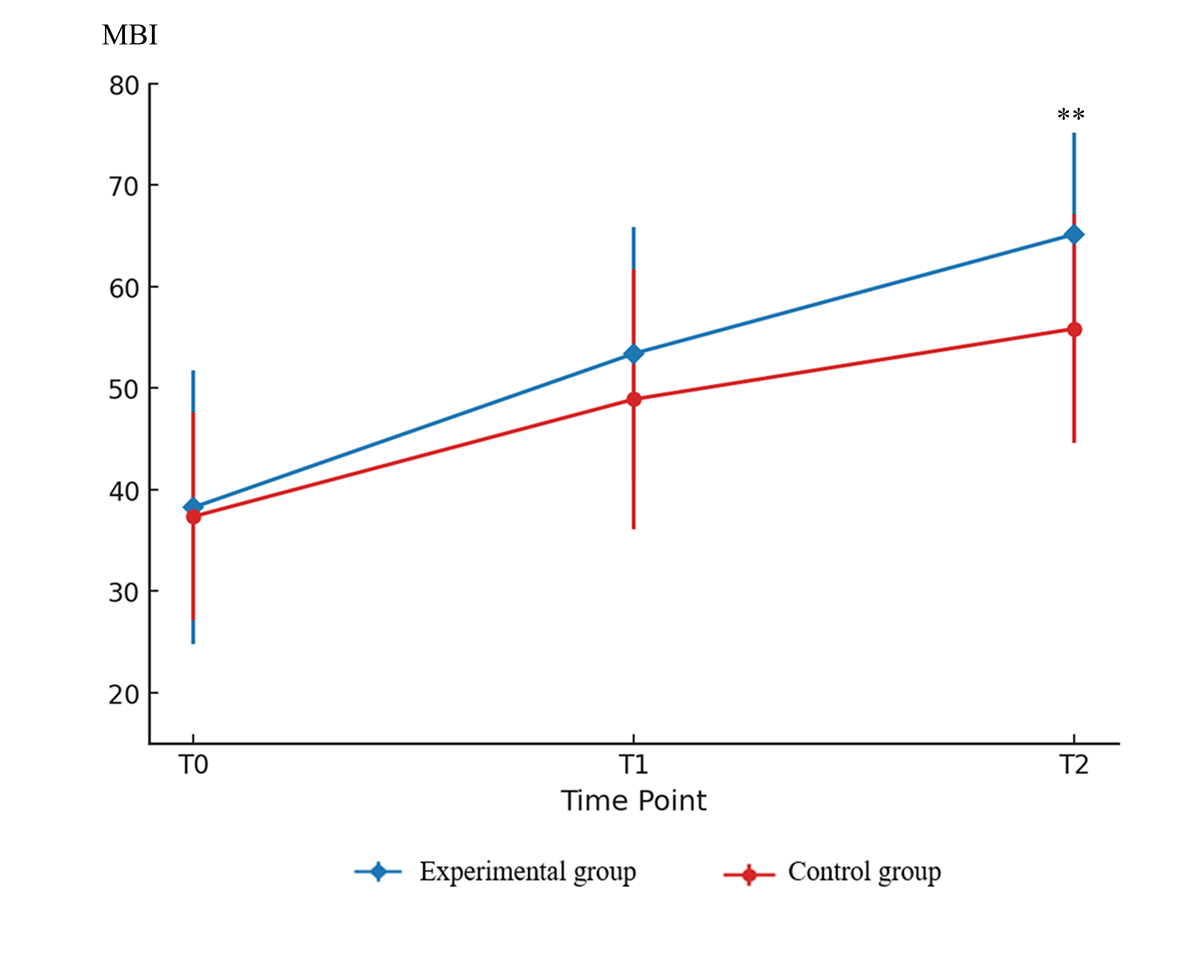
Between-group temporal changes in modified Barthel index. Mean (SD) values are shown at baseline (T0), week 1 (T1), and week 2 (T2). The experimental group exhibited greater improvement than the control group at T2 (**P=.001). MBI: modified Barthel index.

## Discussion

This randomized controlled trial demonstrated that a 2-week, hospital-based CMRTP combined with conventional rehabilitation significantly improved respiratory function and daily functional performance compared with conventional rehabilitation alone in patients with moderate acute stroke. Importantly, no serious adverse events were reported, and the mobile-based platform (AIRHUB) was feasible for implementation in inpatient acute stroke care. To our knowledge, this was the first study to establish the efficacy of a mobile-based respiratory program as a safe and effective adjunct to standard care during the acute phase of stroke.

Compared with prior studies [8,43,44], the experimental group demonstrated comparable improvements in respiratory function, respiratory muscle strength, and daily activities in patients with acute stroke. Choi et al [8] reported that a 4-week comprehensive respiratory muscle training program, incorporating air-stacking and respiratory muscle strengthening, led to significant improvements in FVC and MEP in patients with acute stroke. Similarly, Yoo et al [43] found that delivering the same protocol at the bedside over three weeks resulted in increases in FVC, FEV₁, and MBI. In another study, Wei et al [44] found that a 12-week intervention combining respiratory muscle training, pursed-lip breathing, and diaphragmatic breathing improved FVC, FEV₁, respiratory muscle strength, and MBI in patients with stroke.

FVC, as a comprehensive indicator of ventilatory capacity, was particularly sensitive to changes in respiratory muscle performance [43]. Given the prevalence and clinical relevance of respiratory dysfunction in acute stroke, FVC provided an objective and meaningful measure to evaluate the efficacy of respiratory interventions [45]. In this study, both groups showed improvements in FVC after 2 weeks of intervention, but the experimental group achieved greater gains. These intergroup differences were unlikely to be attributed to spontaneous recovery alone but were more plausibly linked to the structured and synergistic design of the CMRTP, encompassing both muscular and neurological mechanisms.

The observed improvements in respiratory function likely resulted from multilevel adaptations involving both central neural remodeling and peripheral muscular strengthening. The acute poststroke phase represented a critical window of heightened neuroplasticity [46], during which axonal sprouting and synaptic reorganization occur at an accelerated rate [47]. Previous studies showed that exercise can facilitate long-term potentiation–like synaptic plasticity, enhancing functions such as memory and fine motor skills [48,49]. Structured and repetitive respiratory exercises—such as diaphragmatic breathing, pursed-lip breathing, and air-stacking—might elicit similar effects by providing continuous sensory and proprioceptive input to cortical and brainstem respiratory centers, reinforcing sensorimotor pathways and supporting circuit-level functional recovery [50]. In addition to synaptic mechanisms, respiratory training might promote neuroplasticity at the molecular level. Exercise was shown to upregulate the expression of mature brain-derived neurotrophic factor, a key modulator of synaptic strength and corticospinal connectivity [51,52]. These effects might enhance the cortical drive to spinal respiratory motor neurons, facilitating compensatory activation and improving voluntary control over respiratory muscles [53-55].

Peripherally, the CMRTP targeted both inspiratory and expiratory muscle groups through focused protocols. Inspiratory exercises and air-stacking primarily strengthened the diaphragm and intercostal muscles [56], whereas pursed-lip and expiratory resistance breathing enhanced the performance of abdominal and accessory expiratory muscles [57-59]. These interventions likely contributed to the significant improvements in MIP and MEP observed in the intervention group. Repeated mechanical loading and neuromuscular activation might have enhanced motor unit recruitment, firing efficiency, and muscle coordination within the respiratory system [60,61]. Together, these central and peripheral adaptations improved respiratory mechanics and endurance, which might have translated into better exercise tolerance and reduced dyspnea. As a result, participants could engage more effectively in physical rehabilitation and daily activities, reflected in the observed improvements in MBI scores.

Despite these positive findings, no significant between-group differences were observed in FEV₁ and PEF. These indices primarily reflect airway patency and expiratory flow dynamics, which are less responsive to short-term interventions and more sensitive to chronic airway function and expiratory control [62]. The 2-week duration of training may have been insufficient to elicit detectable changes in these parameters. Moreover, poststroke impairment in airway coordination—such as reduced glottic control or weakened expiratory reflexes—may further limit responsiveness to early-phase interventions [63,64]. Longer or more targeted expiratory-focused protocols may be needed to achieve improvements in these measures.

Compared with other motor telerehabilitation studies, our findings highlight the value of a simplified, focused intervention suitable for patients with acute stroke. Previous programs targeting upper limb [65], spinal stability [66], or core function [67] have shown functional gains, but often required long durations (8‐12 wk), complex protocols, or real-time supervision, which may limit adherence or scalability. Some participants in these studies reported training fatigue, reduced engagement, or low compliance [66,67]. In contrast, our 2-week respiratory protocol achieved a high adherence rate (96%) with minimal supervision, supported by an app-based system that provided clear instructions and immediate feedback. Rather than using videoconferencing or immersive visual reality [25,66,68], which required constant therapist involvement, our approach relied on routine caregiver support, enhancing feasibility in clinical settings. By focusing on a single, targeted domain—respiratory recovery—our program provided a practical and scalable model for early-phase telerehabilitation in stroke care.

Despite these promising findings, the results must be interpreted in the context of certain limitations. The intervention period was limited to two weeks due to constraints imposed by the Chinese health insurance system, whereas previous studies suggested that respiratory training typically spans at least 4 weeks to maximize benefit [8,59,69]. To address this, the CMRTP was intentionally designed as a high-intensity, twice-daily, multicomponent protocol targeting multiple facets of respiratory function simultaneously. Although the improvements observed in FVC were smaller than those reported in longer trials—for example, a recent meta-analysis reported an average FVC increase of 0.87 L in early stroke [9]—our findings suggest that a condensed, intensive approach could still yield meaningful gains, particularly for patients in the acute phase of stroke.

The AIRHUB platform significantly facilitated the delivery of this program by overcoming temporal and spatial barriers that often impede rehabilitation in the acute phase. It mitigated treatment disruptions caused by limited clinical staff availability or urgent medical needs, ensuring continuous and stable access to respiratory training. Its user-friendly interface, guided multimedia instructions, and integration with nursing oversight enabled patients to complete high-quality training sessions independently and consistently. The hybrid model, combining digital support with in-hospital supervision, likely contributed to the high adherence rate (97.5%) and absence of adverse events. Compared to home-based rehabilitation models, this approach provided greater control over intervention fidelity, which was critical in early-phase recovery.

Nevertheless, several limitations should be acknowledged. First, although outcome assessors were blinded, patient awareness of group allocation might have introduced performance bias, particularly for effort-dependent measures such as MIP and MEP. Second, the relatively short duration of the intervention limited the interpretation of long-term effects. Third, the modest sample size (N=40), while sufficient to detect primary effects, might reduce generalizability and statistical power to detect smaller but clinically meaningful differences.

Recent advances in respiratory rehabilitation included virtual reality biofeedback systems that visualized and quantified respiratory data, offering more effective and engaging training experiences [70,71]. Devices such as Acapella combined with the active cycle of breathing technique improved lung function in perioperative patients with lung cancer [72], and mobile-based intelligent trainers like AeroFit IMT enhanced respiratory muscle strength without inducing fatigue [73]. While these technologies showed promise, their use often required specialized equipment and supervision, which might limit feasibility in acute stroke care [74]. Therefore, incentive spirometry was chosen in this study for its safety, low cost, ease of training, and visual feedback–facilitated adherence [74,75].

Future studies should investigate longer interventions, larger multicenter cohorts, and the integration of advanced or digitally enhanced respiratory devices. Comparative studies of different training intensities, configurations, and delivery models, including home-based telerehabilitation, will be essential to optimize individualized protocols and evaluate scalability in diverse clinical settings.

This randomized controlled trial demonstrated that a 2-week, hospital-based CMRTP combined with conventional rehabilitation significantly improved respiratory function and daily functional performance in patients with moderate acute stroke. The intervention was safe, well-tolerated, and achieved high adherence, suggesting good feasibility for inpatient application. These findings support the integration of mobile-based respiratory training as an effective adjunct to conventional hospital rehabilitation for early respiratory recovery after stroke. Larger multicenter studies with extended follow-up are needed to confirm the long-term benefits and cost-effectiveness of this approach.

## Supplementary material

10.2196/78637 Multimedia Appendix 1 Detailed intervention protocol and supplementary data tables of outcome measures.

10.2196/78637 Checklist 1 CONSORT checklist.

## Abbreviations

ANCOVA: analysis of covariance
CMRTP: comprehensive mobile-based respiratory training program
FEV₁: forced expiratory volume in 1 second
FVC: forced vital capacity
HIPAA: Health Insurance Portability and Accountability Act
LS: least squares
MBI: modified Barthel index
MEP: maximal expiratory pressure
MIP: maximal inspiratory pressure
NIHSS: National Institutes of Health Stroke Scale
PEF: peak expiratory flow
