# Supplementary material for: Effect of a Comprehensive Mobile-Based Respiratory Training Program on Respiratory Function in Survivors of Acute Stroke: Randomized Controlled Trial
Source: JMIR Mhealth Uhealth. 2026 Apr 14;14:e78637. doi: 10.2196/78637 (PMC13078608; doi:10.2196/78637)
Supplement: Multimedia Appendix 1 [file mhealth-v14-e78637-s001.docx]

**Supplemental Material**

The CTRTP was composed of breathing exercises and respiratory muscle training. The detailed training content was presented below.

**Respiratory exercise**

**Pursed-lip breathing exercise:** The subject is positioned in a semi-recumbent posture with both hips and knees flexed, and the unaffected hand placed on the abdomen. The mouth is closed, and the subject inhales deeply through the nose to maximum capacity for 3 seconds, holds the breath for 3-5 seconds, then purses the lips as if whistling and slowly exhales completely. During inhalation, the unaffected hand should feel the abdomen rise and expand outward, while during exhalation, the abdomen should descend and contract.

**Diaphragmatic breathing exercise:**The subject is positioned in a semi-recumbent posture with both hips and knees flexed, and the unaffected hand placed on the abdomen. The subject inhales calmly, with the unaffected hand following the rise of the abdomen. After completing the inhalation, hold the breath for 1-2 seconds, then exhale slowly through pursed lips, resembling a whistle. While exhaling, the unaffected hand gently presses upward and inward to assist the diaphragm in lifting.

**Stacked breathing exercise：**The subject is positioned in a semi-recumbent posture with both hips and knees flexed, and the body in a relaxed state. Inhale lightly through the nose, then pause briefly. Inhale a second time to expand the chest, pause again, and take a third breath to fill the entire lungs. After completing the inhalation, hold the breath for 3 seconds to allow full lung expansion. Finally, exhale slowly through the mouth, progressively expelling air from the lungs.

**Respiratory muscle training**

**Inspiratory muscle training:** The subject is positioned in a semi-recumbent posture with both hips and knees flexed, and the body in a relaxed state. The unaffected hand holds the breathing training equipment (JiYue Breathing Trainer E-5000cc) (Aomei Medical Int., Ningbo, Zhejiang). After exhaling slightly, the subject inhales slowly to maximum capacity, observing the small ball rise to the target value on the device. The first stage started with 50% of baseline maximum Inspiratory pressure each subject was able to manage(3).After every 6 sessions, the threshold was increased by a further 5 cm H2O. If the subject experienced difficulty in breathing, the pressure was maintained and repeated.

**Expiratory muscle training**: The subject is positioned in a semi-recumbent posture with both hips and knees flexed, and the body in a relaxed state. The unaffected hand grips the breathing training equipment (JiYue Breathing Trainer E-5000cc), inhales fully, holds the breath for 2-3 seconds, then exhales slowly and completely through the mouthpiece, watching the activity valve rise to the target value on the device. The initial threshold was set at 50% of each patient’s baseline maximal expiratory pressure. After every 6 sessions, the threshold was increased by 5 cmH₂O, unless breathing difficulty was reported, in which case the threshold was maintained.

Table S1 The outcome of Mauchly’s test

| **Outcome** | ***Mauchly W*** | ***P* (**Mauchly’s test**)** | **Greenhouse–Geisser ε** |
| --- | --- | --- | --- |
| **FVC (L)** | 0.98 | .68 | 0.98 |
| **FEV1 (L)** | 0.79 | .02* | 0.83 |
| **PEF (L/s)** | 0.79 | .01* | 0.83 |
| **MIP (cmH₂O)** | 0.99 | .86 | 0.99 |
| **MEP (cmH₂O)** | 0.69 | .001* | 0.76 |
| **MBI** | 0.99 | .97 | 0.99 |

Abbreviations: FVC, forced vital capacity; FEV1, forced expiratory volume in 1 second; PEF, peak expiratory flow; MIP, inspiratory pressure; MEP, maximal expiratory pressure; MBI, Modified Barthel Index.

*P＜.05

|  | | **Group Effect** | | | | **Time Effect** | | | **Group*Time Interaction** | | | |  |
| --- | --- | --- | --- | --- | --- | --- | --- | --- | --- | --- | --- | --- | --- |
| **Outcome** | Std. Error | | t value/ df | | P | Std. Error | t value/ df | P | | Std. Error | t value/ df | P |  |
| **FVC (L)** | 0.28 | | 1.69/51.18 | .09 | | 0.07 | 5.89/79.38 | ＜.001* | | 0.09 | 1.71/79.24 | .009* | |
| **FEV1 (L)** | 0.24 | | 0.63/66.45 | .53 | | 0.08 | 4.03/79.73 | ＜.001* | | 0.12 | 1.48/79.45 | .14 | |
| **PEF (L/s)** | 0.35 | | 0.36/60.12 | .71 | | 0.11 | 2.72/79.56 | 0.008* | | 0.16 | 2.13/79.33 | .05 | |
| **MIP (cmH₂O)** | 4.68 | | 2.89/48.02 | .005* | | 1.01 | 4.28/79.27 | ＜.001* | | 1.41 | 3.11/79.17 | .002* | |
| **MEP (cmH₂O)** | 6.18 | | 1.94/52.15 | .047* | | 1.59 | 1.07/79.37 | 0.29 | | 2.24 | 3.47/79.22 | ＜.001* | |
| **MBI (score)** | 3.56 | | 1.56/53.44 | .12 | | 0.98 | 9.67/78.99 | ＜.001* | | 1.37 | 2.93/78.83 | .004* | |

Table S2 Results of mixed linear model analysis

Abbreviations: FVC, forced vital capacity; FEV1, forced expiratory volume in 1 second; PEF, peak expiratory flow; MIP, inspiratory pressure; MEP, maximal expiratory pressure; MBI, Modified Barthel Index.

*P＜.05
